# Acute Kidney Injury in Oncology Patients

**DOI:** 10.7150/jca.45382

**Published:** 2020-05-22

**Authors:** Li-Yan Wang, Jia-Ni Wang, Zong-Li Diao, Yi-Ming Guan, Wen-Hu Liu

**Affiliations:** 1 Department of Nephrology, Beijing Friendship Hospital, Capital Medical University, Beijing 100050, China.; 2 National Cancer Center/ National Clinical Research Center for Cancer/ Cancer Hospital, Chinese Academy of Medical Sciences and Peking Union Medical College, Beijing, 100021, China.

**Keywords:** oncology, renal injury, etiology, pathophysiology, treatment

## Abstract

With rapid progress in cancer diagnosis and treatment in the last two decades, outcomes in oncological patients have improved significantly. However, the incidence of acute kidney injury (AKI) in this population has also increased significantly. AKI complicates many aspects of patients' care and adversely affects their prognoses; thus, accurately diagnosing the risk factors for AKI ensures appropriate management. AKI may be caused by pre-renal, intrinsic renal, and post-renal reasons, as well as for combined reasons. This review summarizes the potential etiologies of AKI according to the three classifications. For each underlying cause of AKI, the cancer itself and/or cancer treatment may contribute to a patient developing AKI. Therefore, we present disease- and treatment-related factors for each cause category, with special focus on immune checkpoint inhibitors, which are being used increasingly more often. It is important for nephrology services to be knowledgeable to provide the best level of care.

## Introduction

Oncology patients are a risk population for developing acute kidney injury (AKI), and the prevalence rate of AKI in oncology populations is 7.5%-9.3% 1,2. AKI increases mortality in oncology patients; therefore, the burden of this disease in patients with cancer is a growing concern. With rapid progress in cancer diagnosis and treatment in the last two decades, outcomes in oncology patients have improved significantly; however, the incidence of AKI has also increased significantly. From 2006 to 2016, cancer incidence increased by 28% worldwide 1. AKI complicates many aspects of patients' care and adversely affects their prognoses. Even traditional risk factors for AKI such as contrast materials, may increase the rate of AKI in oncology patients from 0.3% to 2.3% compared with patients without cancer 3. Newer therapies also contribute to the increased incidence of AKI.

**Figure 1:**
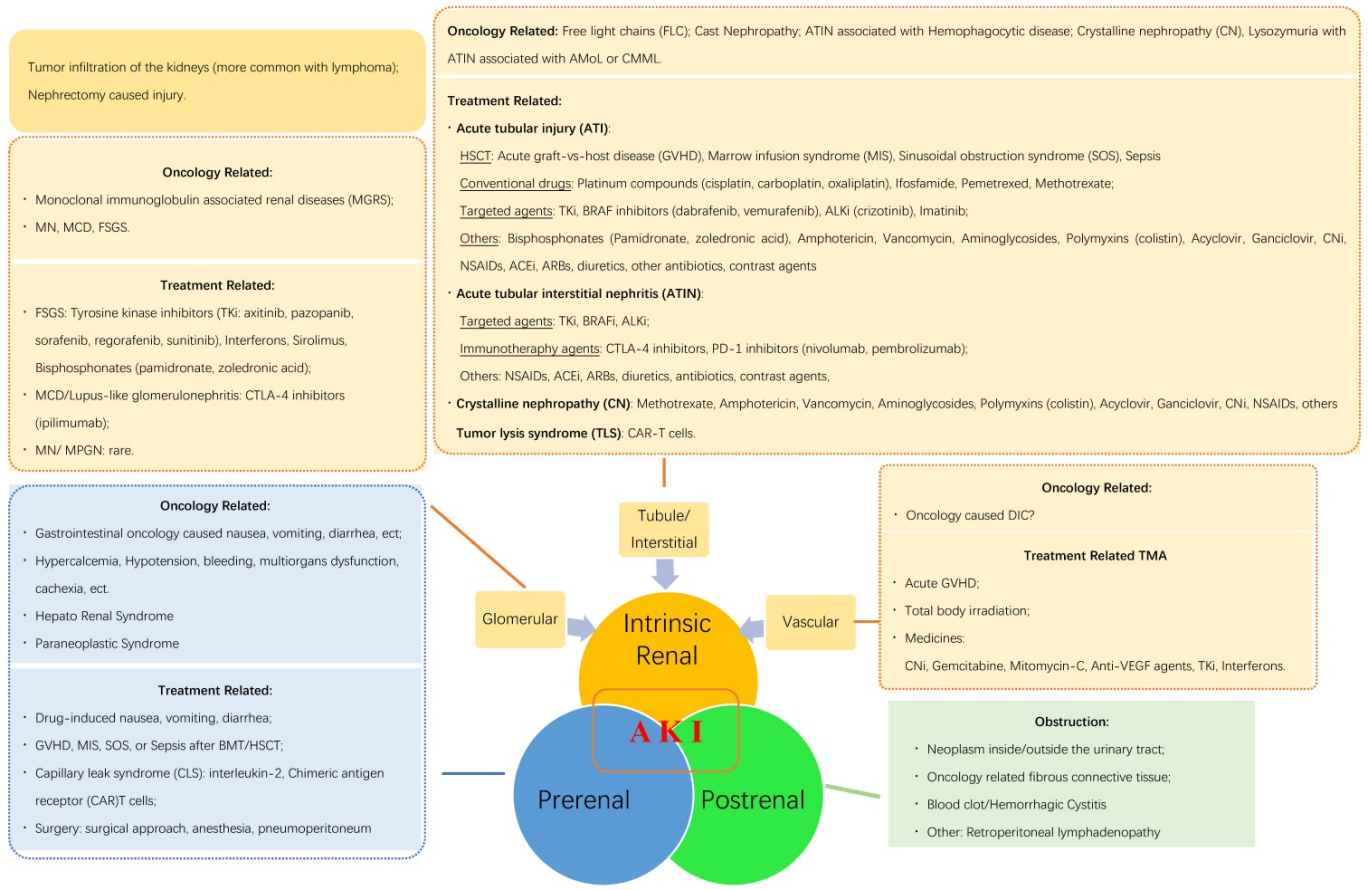
** Etiology of acute kidney injury (AKI) in oncological patients.** AMoL: acute monocytic leukemia; CMML: chronic myelomonocytic leukemia; HCT: hematopoietic stem cell transplantation; ALKi: anaplastic lymphoma kinase inhibitors; CNi: calcineurin inhibitor; NSAID: non-steroidal anti-inflammatory drug; ACEi: angiotensin-converting enzyme inhibitor; ARB: angiotensin receptor blocker; CTLA-4 inhibitors: cytotoxic T-lymphocyte-associated antigen-4 inhibitors; PD-1 inhibitors: programmed death-1 inhibitors; FSGS: focal segmental glomerulosclerosis; MCD: minimal change disease; MN: membrane nephropathy; MPGN: membranous proliferative glomerulonephritis; SOS: sinusoidal obstruction syndrome; DIC: disseminated intravascular coagulation; VEGF: vascular endothelial growth factor; BMT: bone marrow transplantation.

In this review, we summarize the reasons for AKI in oncology patients (**Fig. 1**) to provide useful clinical information. According to the pathophysiological mechanisms and the anatomical injury sites, AKI may be induced by post-renal, pre-renal, and intrinsic renal etiologies. Additionally, cancer itself and/or related treatment factors may induce AKI by each of the three listed mechanisms.

## Post-renal AKI

Most patients with AKI have post-renal causes, and urinary tract obstruction (UTO) is the major cause of post-renal AKI; anuria or oliguria quickly follow UTO. Imaging using ultrasonography, X-ray, magnetic resonance imaging, or computed tomography provide typical UTO images to support the diagnosis of post-renal AKI in most instances. Urinary system carcinoma, metastatic cancer, enlarged lymph nodes, and retroperitoneal fibrous connective tissue all can induce UTO 4.

In some patients, blood clots produced by bleeding from neoplastic tissues or hemorrhagic cystitis induced by drugs can also cause UTO. Polyomavirus hominis type 1 (BK) virus-associated hemorrhagic cystitis (BK-HC) is another common complication after allogeneic hematopoietic stem cell transplantation (allo-HSCT). The incidence of BK-HC ranges from 7% to 70%, with severe hematuria in 8-27% of patients receiving allo-HSCT 5. Massive hematuria may lead to UTO because of clots and urinary retention, causing post-renal AKI 6. Diagnosis is confirmed by quantitatively measuring viral copies in plasma or urine. It has been shown that being an asymptomatic carrier of BK virus after HSCT is a risk factor for AKI, especially when viremia is detectable 7.

Once the UTO is relieved, patients may recover rapidly from AKI, even in patients with chronic kidney disease (CKD), which may also be caused by UTO. If patients have several factors inducing AKI, post-renal factors should be treated first. Otherwise, it is difficult to identify other etiological factors.

## Pre-renal AKI

### Cancer-related reasons

Renal ischemia is a core mechanism in pre-renal AKI. Gastrointestinal symptoms associated with oncology, such as nausea, vomiting, and diarrhea, induce decreased food intake, malnutrition, and even cachexia, and then cause hypotension, low blood volume, and low renal perfusion. Cancer also causes bleeding, tumor thrombus, hepatorenal syndrome in hepatic carcinomas, paraneoplastic syndrome, hypercalcemia, and nephrectomy-induced ischemic injury, which can also contribute to AKI.

### Treatment-related reasons

Drug-induced gastrointestinal effects are common. Many routine agents, such as diuretics, angiotensin receptor-blockers, and angiotensin-converting enzyme inhibitors, are well-known pre-renal risk factors for AKI. Several additional treatment-related complications have been reported recently, such as bone marrow transplantation or HSCT-induced graft-vs-host disease (GVHD), marrow infusion syndrome, and veno-occlusive disease (VOD) or sinusoidal obstruction syndrome, and immunotherapeutic agents such as interleukin-2 (IL-2) and chimeric antigen receptor T cell-induced capillary leak syndrome (CLS), which can all cause AKI associated with renal hypoperfusion.

AKI is a common comorbidity in pediatric patients following HSCT, with an incidence ranging from 11% to 84%. Of these, 5%-10% of patients may require renal replacement therapy (RRT) 8,9. Pre-renal AKI may occur at any time during and after HSCT. This may be a result of fluid loss caused by chemotherapy-induced vomiting or diarrhea, iatrogenic excessive diuresis, and tumor lysis syndrome (TLS) when remission was not achieved before conditioning 10. Two other complications causing AKI in the early post-transplant period are CLS and engraftment syndrome. Both are caused by the release of pro-inflammatory cytokines and may manifest after auto-HSCT and allo-HSCT as fluid retention and non-infectious fever 11. CLS often presents within 2 weeks post-HSCT as peripheral edema and serosal effusion resistant to diuretics 6. CLS as a component of cardiorenal syndrome (CRS) deserves special attention in the field of haploidentical HSCT. In this instance, CRS complicates 87% of cases, and 12% of patients experience severe CRS, which correlates with markedly higher mortality; clinically significant renal failure was found in 14% of patients with severe CRS. Treatment options are steroids and currently-investigated anti-IL-6 therapies 12. Engraftment syndrome presents similarly to CLS but occurs mainly during neutrophil regeneration and is often accompanied by fever and rash with multiorgan dysfunction. Kidney dysfunction in engraftment syndrome was found in 8% of allo-HSCT recipients 13 and in 27% of auto-HSCT recipients 14. Engraftment syndrome responds well to glucocorticoid treatment, provided that the treatment is implemented early 13.

VOD/sinusoidal obstruction syndrome is a serious complication of HSCT, with mortality in its severe form exceeding 80% 15. The reported incidence of VOD varies widely. In patients undergoing traditional myeloablative conditioning allo-HSCT, approximately 14% of patients develop VOD 15. The mechanism of kidney injury in VOD is analogous to the mechanism in hepatorenal syndrome 15, in that endothelial damage and coagulation results in obstruction of hepatic sinuses and leads to portal hypertension 16. Kidney specimens typically show no structural changes, which indicates renal hypoperfusion secondary to splanchnic vasodilatation and decreased effective blood volume 17. Managing fluid balance is critical in patients receiving HSCT. In particular, sodium and fluid restriction and diuretics are cornerstones to prevent fluid overload in VOD-associated AKI 18. Continuous RRT has become a key treatment for critically-ill patients with AKI and fluid overload. The use of a standardized, evidence-based fluid balance protocols and early initiation of continuous RRT for HSCT-related AKI is associated with good outcomes 8, and initiating earlier therapy with defibrotide improves survival 19.

Pre-operative dehydration may be associated with post-operative AKI. Considering nephrectomy, where a substantial amount of functional parenchyma is being removed; the clinical significance of AKI is difficult to gauge. Patients who were dehydrated and mildly dehydrated had an increased risk of AKI (odds ratio (OR) = 4.1, and OR = 2.4, respectively) compared with euhydrated patients, in one study 20. Pre-operative fasting and anesthesia coupled with intra-operative fluid losses, diminished cardiac output, and pneumoperitoneum can also significantly reduce kidney perfusion and subsequently lead to AKI 20. Liberal peri-operative administration of intravenous crystalloids may reduce the risk of post-operative AKI, as noted in a recent randomized controlled trial 21.

## Intrinsic AKI

Intrinsic renal injury is the major reason for AKI in oncology patients. Both nephrectomy and tumor infiltration of the kidneys directly decrease kidney functional mass and cause structural changes in the residual renal tissue, which damage kidney function. In oncological patients not undergoing nephrectomy and with no renal infiltration, considering the anatomical sites of injury, acute tubular necrosis (ATN) is the most common etiology of AKI. The incidences of glomerular nephropathy and microvascular diseases have increased recently in these patients.

### Glomerular Injury

Glomerular injury may be induced by native cell damage with paraneoplastic glomerular disorders or monoclonal gammopathy of renal significance. Many oncological drugs can also induce different types of glomerular nephritis.

#### Cancer-associated glomerular nephritis

Several glomerular diseases are associated with hematological tumors. In chronic lymphocytic leukemia (CLL), the most frequently reported findings are membrane proliferation glomerular nephritis (MPGN, 36%) and membrane nephropathy (19%) 4. Other rare findings are minimal change disease (MCD), immunotactoid glomerulopathy, and focal segmental glomerular sclerosis (FSGS) 22,23. However, most cases of MPGN included direct deposition of monoclonal proteins in 2.5%-60% of patients, in the form of proliferative glomerular nephritis, C3 deposits, or cryoglobulinemia 22-24. Abnormal serum free light chains can be detected in 30%-40% of patients with CLL 4. The monoclonal protein secreted by the B-cell clone can be either directly involved in the pathogenesis of the lesions, as in cases of fibrillary glomerulopathy, immunotactoid nephropathy, amyloid light chain amyloidosis, or type I/II cryoglobulinemia, or indirectly in cases of MPGN not related to cryoglobulinemia 23. However, glomerular nephritis is uncommon in patients with multiple myeloma. Most cases of nephrotic syndrome in patients with multiple myeloma are related to amyloid deposition and rarely to glomerular nephritis. FSGS associated with hematological tumors is infrequent, and when it occurs, it is unclear whether FSGS is related to the cancer or a coincidence. A possible explanation for the association between FSGS and hematological tumors is that vascular endothelial growth factor and heparanase have been reported to alter glomerular permeability in patients with FSGS 25. These causes of glomerular nephritis can contribute to AKI either independently, or combined with other risk factors.

#### Treatment-associated glomerular nephritis

Tyrosine kinase inhibitors (TKIs) such as axitinib, pazopanib, sorafenib, regorafenib, and sunitinib, as well as interferons, sirolimus, bisphosphonates (pamidronate and zoledronic acid) can induce FSGS. Cytotoxic T-lymphocyte-associated antigen (CTLA)-4 inhibitors such as ipilimumab, can induce MCD or lupus-like glomerular nephritis 26,27. Other kidney manifestations in patients receiving checkpoint inhibitors (CPIs) are lupus nephropathy, pauci-immune glomerular nephritis, immunoglobulin A (IgA) nephropathy, as well as nephrotic syndrome with FSGS, MCD, and membrane nephropathy 28. However, AKI caused by these treatments secondary to glomerular nephritis is rare.

Apart from the obvious risk of pre-renal AKI in the course of gastrointestinal GVHD, moderate and severe presentations of GVHD are correlated with AKI. Evidence suggests that glomerular nephritis and proteinuria are associated with GVHD 29. Undoubtedly, renal damage in GVHD is propelled by excessive release of pro-inflammatory cytokines such as tumor necrosis factor-α, IL-6, and transforming growth factor-β 29. Evidence for a direct cytotoxic response against kidney tissue in GVHD is missing because kidney biopsies are rarely performed in this group of patients.

### Tubular-interstitial injury

#### Oncology-related reasons

Cancer itself induces tubular-interstitial injury in patients with extremely high serum free light chains levels, cast nephropathy, hemophagocytic disease, crystalline nephropathy, lysozymuria associated with acute monocytic leukemia, or chronic myelomonocytic leukemia. Until 2014, monoclonal gammopathies were classified as monoclonal gammopathy of undetermined significance, smoldering myeloma, symptomatic multiple myeloma, solitary plasmacytoma, and immunoglobulin light chain amyloidosis 30. In patients with smoldering myeloma and multiple myeloma, high monoclonal free light chain levels can overwhelm the reabsorption capacity of the proximal tubule so that these chains reach the distal tubule where they interact with Tamm-Horsfall protein, which leads to the generation of myeloma casts. These casts may block glomerular flow and cause tubular atrophy and interstitial fibrosis 31. Myeloma casts have also been associated with Waldenström macroglobulinemia, other lymphomas, and CLL 22. Not all monoclonal free light chains are equally nephrotoxic. The interstitial inflammation is believed to be triggered by the leakage of free light chains into the interstitium. Such inflammation can lead to irreversible kidney injury and scarring. Patients with cast nephropathy typically present with severe AKI, often requiring dialysis 32, and the cast proteins are typically resistant to proteolysis 4. Therefore, recovery of renal function depends on early initiation of chemotherapeutic agents and other supportive measures to reduce ongoing damage from free light chains. In a recent paper 33, the prevalence of renal impairment in a large group of patients with smoldering myeloma (n = 1135) was 20%, at presentation; however, renal function in a large proportion of the patients (54%) improved after anti-myeloma induction therapy.

Vial et al 34 reported three patients with AKI among 10 patients with CLL who underwent renal biopsy over 16 years, including one with interstitial monoclonal lymphoid infiltration. The mechanism of AKI with CLL infiltration is not clearly established but has been hypothesized to involve tubular/microvascular compression causing intra-renal obstruction in addition to an infiltration-associated inflammatory/cytokine response. Granulomatous interstitial nephritis (non-infection related) in patients with CLL has been described in case reports and small case series 35, with partial recovery in 90% of the patients receiving treatment for CLL. Ouyang et al demonstrated a rare case of rhabdomyolysis causing T-cell lymphoma multi-organ dysfunction, including AKI related to myoglobulin nephrotoxin 32.

#### Treatment-related reasons

As in other patients, ATN caused by nephrotoxic drugs is common in oncology patients. Well-known drugs include antibiotics (especially amphotericin, vancomycin, aminoglycosides, and polymyxins), acyclovir, ganciclovir, bisphosphonates, calcineurin inhibitors (CNIs), and contrast materials. Conventional cancer drugs such as platinum compounds, ifosfamide, pemetrexed, and methotrexate, as well as targeted agents such as TKIs, anaplastic lymphoma kinase inhibitors, and BRAF gene inhibitors induce ATN. In some patients, acute tubular interstitial nephritis (ATIN) may appear after using either targeted drugs or standard agents. Immunotherapeutic agents such as CTLA-4 inhibitors and programmed death (PD)-1 inhibitors (nivolumab, pembrolizumab) can also induce ATIN.

CPIs are attracting attention as novel cancer therapeutic agents. AKI with the histological feature of ATIN is a severe and common condition in CPI-associated renal adverse events 36,37. CPIs have two main targets: CTLA-4 and PD-1 and its ligand, PD-L1 38. The anti-CTLA-4 monoclonal antibody ipilimumab, anti-PD-1 antibodies nivolumab and pembrolizumab, and the anti-PD-L1 antibodies atezolizumab and durvalumab are considered to reactivate cytotoxic T cells, leading to tumor cell lysis by blunting the braking mechanisms of the immune system 38. Since 2014 39,40, with the first report of ipilimumab-induced granulomatous ATIN, the number of reported cases of CPI-induced AKI has been increasing. The manufacturer states that immune-mediated nephritis and renal dysfunction occurred in 1.2% (23/1994) to 1.7% (5/287) of patients receiving nivolumab as a single agent 41. Mamlouk et al 28 showed that ATIN was the most common pathological finding on biopsy (14/16) and was present in almost all cases as either the major microscopic finding or as a mild form of interstitial inflammation in association with other glomerular pathologies (pauci-immune glomerulonephritis, membranous glomerular nephritis, C3 glomerular nephritis, IgA nephropathy, or amyloid A amyloidosis). Immunotherapy-related acute interstitial nephritis could be secondary to the loss of tolerance of drug-specific effector T cells or the development of auto-immunity to kidney auto-antigens after the loss of self-tolerance and potentiation of antigen recognition after blocking the CTLA-4 or PD-1 pathway 42. Discontinuing CPIs and using steroids and further immunosuppression achieved complete to partial renal recovery in these patients 28. A case of nivolumab-induced acute granulomatous TIN in a patient with gastric cancer has been reported. Treatment with methylprednisolone (1.0 mg/kg daily) led to rapid improvement in renal function and decreased numbers of circulating cluster of differentiation 4-positive T cells 43.

TLS results from rapid release of the intracellular contents of dying cancer cells into the bloodstream either spontaneously or in response to cancer therapy. TLS is the most common oncological emergency, with an incidence as high as 26% in high-grade B-cell acute lymphoblastic leukemia 44. The use of venetoclax, a recently approved B-cell lymphoma-2 protein inhibitor for use in a select group of patients with relapsed CLL positive for del17p (high risk of progression) led to drug-induced TLS in 3%-6% of patients 45. Some scholars inferred that thermal ablation could cause oliguric AKI, myoglobinuria, and TLS. Ding et al 46 reported that 23.6% of patients had AKI after microwave ablation of large liver tumors (≥ 5 cm), among whom, 89.4% had non-oliguric AKI, and 10.6% had oliguric AKI.

BK virus and adenovirus deserve special attention regarding kidney injury. Both viruses are found in asymptomatic carriers, and immunosuppression may cause their reactivation in HSCT recipients, leading to HC and, less commonly, AIN 47. While cidofovir may be an efficacious therapy for BK-HC, with 60%-100% response rates according to systematic reviews, the rates of nephrotoxic adverse events is significant (9%-50%) 48. Massive hematuria, flank pain, fever and AKI, as well as high viral load, are suggestive of nephritis, which may be confirmed by renal biopsy, and guide decisions toward more aggressive treatment with cidofovir or leflunomide 49,50.

### Vascular injury

#### Kidney vascular injury associated with oncology

Thrombotic microangiopathy (TMA) is the most common syndrome in kidney vascular injury 51, and disseminated intravascular coagulation (DIC) secondary to cancer may be another reason for AKI. TMA has been reported in patients with CLL. In a study by Strati et al 22, six patients presented with TMA (renal-limited or systemic) as the renal manifestation of CLL. All six patients had clinical signs of AKI, proteinuria, hemolysis, anemia, elevated lactate dehydrogenase (LDH) levels, the presence of schistocytes on peripheral blood smears, and low haptoglobin. Four of the six patients responded to treatment for CLL; one required HSCT, and the other had concurrent lung cancer 22.

#### TMA associated with oncological treatments

TMA develops mainly after oncology-related treatments such as treatments for viral infections (cytomegalovirus, adenovirus) and acute GVHD, following total body irradiation or VOD/sinusoidal obstruction syndrome, and treatment with drugs such as CNIs, gemcitabine, mitomycin-C, anti-vascular endothelial growth factor agents, TKI, and interferons. Kidneys are affected most often, and autopsy studies also confirm the features of TMA in patients with normal creatinine levels 52.

Since the first case of gemcitabine-associated TMA (G-TMA) was reported in 1994 53, many case reports have been published, with an estimated incidence of 1.4% 54. Although rare, G-TMA is a severe complication and is associated with notable mortality and renal damage that may lead to end-stage renal disease (ESRD). A French cohort study 55 of 120 patients with G-TMA reported a rate of AKI of 97.4%, and 27.8% of these patients required dialysis. G-TMA can occur earlier, especially if gemcitabine is used with other drugs such as pegylated liposomal doxorubicin 56, tegafur 57, and oxaliplatin 58, or if several lines of chemotherapy preceded gemcitabine 59. The exact pathophysiology of G-TMA is unknown. The monoclonal antibody targeting complement factor C5, eculizumab, has been prescribed in some patients with G-TMA with good outcomes, based on a possible role of complement alternative pathway dysregulation 55,60. Pazopanib is a TKI that limits tumor growth by inhibiting growth factors and resulting in inhibition of angiogenesis. TMA occurred in 7/977 patients during phase 3 trials of pazopanib 61. Of these patients, six had TMA diagnosed within 90 days of treatment initiation, and all patients experienced resolution with discontinuation of the drug.

Transplant-associated TMA is a complex and not fully understood disorder that resembles atypical hemolytic uremic syndrome. It is thought that damage to the vascular endothelium triggers platelet aggregation in the microvasculature and excess activation of complement, which leads to thrombosis and fibrin deposition 62. Development of TMA is correlated with lower overall survival in transplant recipients (approximately 30% during the first year), and the mortality rate in patients with TMA with multiorgan involvement is reported to be as high as 90%; therefore, new therapeutic approaches are urgently needed 52. Treatment of TMA is based on aggressive management of the triggering factors and supportive care. When managing hypertension, calcium blockers are considered the safest option for injured kidneys, while diuretics are the drugs of choice to manage fluid retention 63. There is no universally-approved approach to the specific treatment of TMA. The Evidence-Based Medicine Trial recommends replacing CNIs in GVHD prophylaxis with other immunosuppressive agents as an initial step 6. Plasmapheresis is another therapeutic option, the early use of which, combined with CNI withdrawal, provides a 27%-80% response rate. Several case reports demonstrated good effects of plasmapheresis combined with rituximab in patients with antibodies against complement factor H.

## Multifactorial AKI

Clinically, AKI develops for several reasons. Multiple pathophysiological mechanisms may exist concurrently or develop subsequently.

Sepsis is characterized by a combination of factors, both hemodynamic and inflammatory, that coalesce to cause AKI. Oncology patients are at risk of developing sepsis; many are immunocompromised because of the cancer, itself, and secondary to anti-cancer therapies. Oncology patients are also at risk of microbiome disruption secondary to cancer treatment and antibiotics. Therefore, early diagnosis and treatment of infections is crucial to prevent AKI. In cases of sepsis-associated AKI, optimizing fluid therapy and withdrawing nephrotoxic drugs are essential. Additionally, early initiation of RRT before the development of fluid overload may improve treatment outcomes 43.

Establishing a diagnosis of drug nephrotoxicity may be challenging in oncology patients treated with numerous agents. In addition to their immediate toxic effects on the renal parenchyma, these agents might decrease renal functional reserve 64 when administered repeatedly, and thus, make the kidneys more sensitive to further sources of injury, namely, sepsis in the intensive care unit (ICU). The growing development and use of targeted therapies, which are associated with renal toxicity 64, might amplify this issue in the future. Medications most often associated with TIN are CNIs, antibiotics, and proton pump inhibitors 65. Importantly, despite a proven nephroprotective role of antagonists of the renin-angiotensin-aldosterone system (RAAS), these drugs may cause harm instead of good when used in patients with dehydration, hypotension, hyperkalemia, progressing AKI, and advanced CKD, as well as in combination with non-steroidal anti-inflammatory drugs (NSAIDs). Recent studies noted an increased risk of AKI in patients with sepsis treated with renin-angiotensin-aldosterone antagonists 66.

Long pre-operative fasting periods, water deprivation, and loss of body fluids caused by long and intensive intra-operative thermal ablation can lead to a variety of pathophysiological changes affecting the glomerular filtration rate, such as hypovolemia, increased blood viscosity, increased destruction of blood cell components, acid-base imbalances, damage to renal microvascular endothelial cells, and the formation of urinary casts, which eventually lead to oliguric AKI 46.

## Risk factors, preventive measures, and treatments for AKI in oncology patients

The likelihood of developing AKI depends not only on the severity of the patient's condition but also on the type of cancer and anti-cancer therapy. In one cohort study from China 1, the total incidence of AKI was 7.5% in 136 756 oncology patients, in whom the top three primary carcinomas were bladder cancer, leukemia, and lymphoma. In another study of 163 071 oncology patients in Canada 2, the cumulative incidence of AKI was 9.3%. The top three cancers with AKI were myeloma (26%), bladder cancer (19.0%), and leukemia (15.4%). In ICU's, the rate of AKI in patients with solid tumors was less than in patients with hematological malignances 49. Seventy-five percent of patients receiving transplants who required treatment in an ICU developed AKI, and among the patients who received RRT, the mortality rate was 95% 10. Therefore, routine screening for AKI should be performed more frequently in patients with hematological malignances or urinary system cancers, and in transplant patients. The incidence of AKI was 59% in a study of 204 patients with solid tumors admitted to ICUs, chiefly related to sepsis (80%), hypovolemia (40%), and outflow tract obstruction (17%) 49. These results indicate the need to prevent infections, and that maintaining proper blood volume and hemodynamic stability are key points in preventing AKI in oncology patients.

Several studies focused on the risk factors for AKI in different types of cancer in patients with baseline CKD or cardiovascular disease 67, namely, anemia 9,46,50, low serum albumin levels 46, different tumor size 68, preoperative dehydration 9,46,50, intra-operative bleeding, and operation time 50. Independent predictors of AKI in the ICU were the simplified acute physiological score II (OR = 1.05), abdominal or pelvic cancer (OR = 2.84), nephrotoxic chemotherapy within the previous 3 months (OR = 3.84), and sepsis (OR = 2.74) 49. The risk of post-operative AKI was greater after radical nephrectomy, and is secondary to a larger amount of functional parenchyma being removed. Patients with prostate cancer are a high-risk group for post-operative AKI because of their older age, tendency to develop obstructive uropathy, and higher risk of post-operative complications such as bleeding and UTO 69.

The most common causes of AKI in patients with hematological malignancies are hypoperfusion, TLS, hemophagocytic syndrome, direct infiltration of malignant cells, and infections 4. Different anti-cancer treatments have different associated prevalences of AKI. Ofatumumab, alemtuzumab, and ibrutinib were the top three offenders, with AKI as the most commonly reported finding, followed by TLS and hyponatremia. The presence of renal dysfunction in patients with multiple myeloma is believed to be secondary to hypercalcemia, recurrent urinary tract infections, renoliths, urate nephropathy, analgesic nephropathy, crystalline nephropathy, and amyloidosis 70. In some patients, these pathophysiological mechanisms appear concurrently, or proceed linearly.

When treating AKI in oncology patients, it is necessary to comprehensively evaluate patients' general condition and identify all factors that may affect renal function, including cancer and non-cancer related factors. Compared with the general population, treating AKI in oncology patients requires more attention. Maintaining adequate hydration is the most important and relatively easy intervention, both for pre-renal AKI and ATN. Timely imaging examinations and disease management in patients with prostate, abdominal, or pelvic cancer are very important because these patients constitute a high-risk group for post-renal AKI. Infections increase morbidity in patients with AKI, so it is also important to identify related symptoms early and begin appropriate therapy. It is necessary to consider that in certain circumstances, treatment can lead to further kidney complications, including AKI. This change in kidney function can necessitate adjusting patients' cancer care, such as chemotherapy options, options for diagnostic evaluation, and other supportive care.

## Conclusion

While major therapeutic advances in the past decade have unquestionably resulted in extended survival, the repeated administration of anti-cancer treatments increases the risk of developing meaningful complications. Ongoing research regarding the early markers of AKI and the use of drugs with less nephrotoxicity may translate into clinical practice in the foreseeable future.
